# Rice yield prediction through integration of biophysical parameters with SAR and optical remote sensing data using machine learning models

**DOI:** 10.1038/s41598-024-72624-4

**Published:** 2024-09-17

**Authors:** Sonam Sah, Dipanwita Haldar, RN Singh, B. Das, Ajeet Singh Nain

**Affiliations:** 1 G. B. Pant University of Agriculture and Technology, Pantnagar, Uttarakhand India; 2 ICAR-National Institute of Abiotic Stress Management, Pune, Maharashtra India; 3 Indian Institute of Remote Sensing, Dehradun, Uttarakhand India; 4 ICAR-Central Coastal Agricultural Research Institute, Goa, Old Goa India

**Keywords:** Remote sensing, LAI, Biomass, Moisture content, XGB, Cubist, Agroecology, Biophysics, Imaging

## Abstract

In an era marked by growing global population and climate variability, ensuring food security has become a paramount concern. Rice, being a staple crop for billions of people, requires accurate and timely yield prediction to ensure global food security. This study was undertaken across two rice crop seasons in the Udham Singh Nagar district of Uttarakhand state to predict rice yield at 45, 60 and 90 days after transplanting (DAT) through machine learning (ML) models, utilizing a combination of optical and Synthetic Aperture Radar (SAR) data in conjunction with crop biophysical parameters. Results revealed that the ML models were able to provide relatively accurate early yield estimates. For summer rice, eXtreme gradient boosting (XGB) was the best-performing model at all three stages (45, 60, and 90 DAT), while for *kharif* rice, the best-performing models at 45, 60, and 90 DAT were XGB, Neural network (NNET), and Cubist, respectively. The combined ranking of ML models showed that prediction accuracy improved as the prediction date approaches harvest, and the best prediction of yield was observed at 90 DAT for both summer and *kharif* rice. Overall rankings indicate that for summer rice, the top three models were XGB, NNET, and Support vector regression, while for *kharif* rice, these were Cubist, NNET, and Random Forest, respectively. The findings of this study offer valuable insights into the potential of the combined use of remote sensing and biophysical parameters using ML models, which enhances food security planning and resource management by enabling more informed decision-making by stakeholders such as farmers, policy planners as well as researchers.

**Supplementary Information:**

The online version contains supplementary material available at 10.1038/s41598-024-72624-4.

## Introduction

Rice, one of the world’s important cereal crops, holds a central position in global agriculture due to its unparalleled significance in feeding a substantial portion of the world’s population^1^. With over half of the worldwide population relying on rice as a dietary staple, its cultivation and production have far-reaching implications for food security, socio-economic stability, and agricultural sustainability worldwide^2^. The importance of rice in world agriculture is most evident in its role as a primary source of nutrition for billions of people, particularly in Asia, where majority of the world’s rice is grown and consumed. This cereal grain provides essential carbohydrates, energy, and vital nutrients to millions of households, making it an indispensable food source. Rice is not only a vital food source but also a driving force in global economies^3^. Its cultivation employs millions of people in farming, processing, and distribution sectors, thus contributing significantly to employment and livelihoods. Additionally, rice is a cornerstone of international trade, with many nations relying on its exports and imports to maintain economic stability and meet domestic demand. Furthermore, the cultivation of rice has environmental implications, as it influences land use, water resources, and ecosystem dynamics.

Early yield predictions of rice is a crucial task for farmers and agricultural industry as it allows for accurate forecasting of crop production and helps in making informed decisions related to planting, fertilization, irrigation, and harvest^4,5^. Integrating remote sensing data with ground-based measurements of biophysical parameters such as leaf area index (LAI), biomass, and plant moisture content, can provide better yield predictions of rice across different growth stages and environmental conditions^6,7^. This not only enhances agricultural productivity and sustainability but also contributes to food security and livelihood improvement for millions of people dependent on rice cultivation worldwide. Accurate yield prediction can also improve rice crop productivity, increase income, and reduce waste by identifying the best agricultural practices, weather conditions and technologies^5,8^.

Traditional methods for predicting crop yields typically involve statistical techniques and crop growth models. Still, these methods have limitations in accounting for varying biotic and abiotic factors and require a lot of data and expertise to use^9^. Additionally, traditional yield predictions have relied on ground-based observations and historical data, often resulting in delayed or inaccurate predictions. However, the advent of remote sensing technology, particularly optical and Synthetic Aperture Radar (SAR) sensors aboard satellites, has revolutionized the way we monitor and forecast rice yields^10^. Several studies have used optical remote sensing indices to predict crop yield^11–14^. Recently, few researchers attempted to predict yield using SAR data in different crops like rice^15^, pearl millet^16^, soybean^17^ and wheat^18^. The fusion of optical and SAR remote sensing data provides a holistic perspective on rice fields^19^. Optical sensors capture information related to vegetation health, canopy structure, and spectral properties, while SAR sensors penetrate cloud cover and provide insights into soil moisture, surface roughness, and crop structure^20^.

Recent advancements in machine learning have also led to improved yield predictions as it can handle complex relationships and large amounts of data from various sources for more accurate and efficient results. Integrating remote sensing data with ground-based measurements of biophysical parameters such as LAI, biomass, and plant moisture content can provide accurate crop yield prediction across different growth stages and environmental conditions^6,7^ and facilitates the development of accurate and timely monitoring systems. Several researchers have utilized machine learning for predicting yield in different crops using satellite data and machine learning models like apple^21^, wheat^22^, soybean^23^, potato^24^, almond^25^, maize^26^, sugarcane^27^. Similarly, machine learning has also been utilized in various studies to predict rice yield by taking remote sensing data^28–30^ or other ancillary/historical data as inputs^31,32^. Researchers have also integrated optical and SAR remote sensing indices in machine learning to predict rice yield^19,33^. Studies that utilized SAR data to predict crop yield reported acceptable accuracies^15^, while others predicting crop yield with synergistic use of optical and SAR data^33–35^ advocated that model performance improves using multi-sensor data as compared to the single sensor. This was mainly because of cloud interference in optical data, which is compensated by using SAR data. However, there is no literature on predicting rice yield by combining optical data, SAR data and ground-based biophysical parameters of the crop.

Against this backdrop, the study aimed to predict summer and *kharif* rice crop yields at three growth stages using ML models that integrate both biophysical and remote sensing parameters. This approach advances our understanding of the complex interactions between crop development and remote sensing data, offering valuable insights for improving yield prediction accuracy.

## Materials and methods

### Study area

**Fig. 1:**
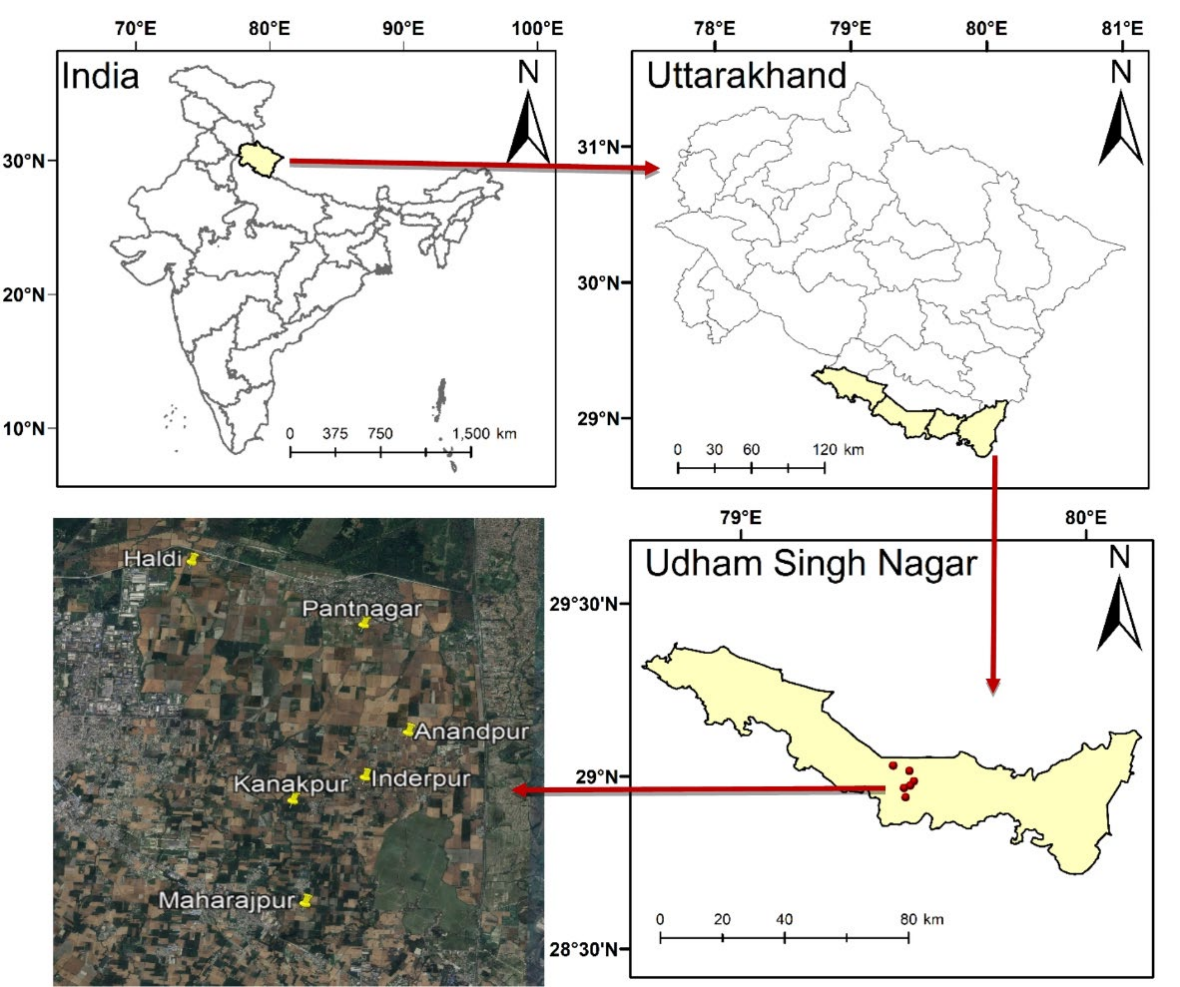
Map displaying the study area and the locations of villages from which data were collected. Created by ArcGIS Desktop v 9.1. https://www.esri.com/en-us/arcgis/products/arcgis-desktop/overview.

The study was carried out in the Udham Singh Nagar district of Uttarakhand during both the summer and *kharif* seasons of 2021. Situated within the “*Terai*” belt, the study area rests in the foothills of the Shivalik range of the Himalayas (Fig. 1).

### Ground truth data

The timings of ground truth data collection were synchronized with satellite passes during both the summer and *kharif* seasons of 2021. Data collection was distributed throughout the study area. The data were gathered from agricultural farms in Govind Ballabh Pant University of Agriculture and Technology (GBPUAT), Pantnagar and from various farmers’ fields located in Pantnagar, Haldi, Kanakpur, Maharajpur, Inderpur, and Anandpur villages within the Udham Singh Nagar district of Uttarakhand, India (Fig. 1). The collected data consists of field latitude, longitude, crop stage, and crop biophysical parameters.

### Crop biophysical data

The biophysical characteristics of the rice crop were observed at three significant growth phases, specifically at 45, 60, and 90 Days After Transplanting (DAT). The six biophysical parameters used in the study include fresh biomass (FBM) in t/ha, dry biomass (DBM) in t/ha, plant moisture content (MC) in %, leaf area index (LAI), plant height in cm and yield in t/ha. DBM was obtained after oven-drying the plant samples. For recording LAI, plant canopy analyser model PCA-VH-1-101 of Virtual Hydromet was used.

### Remote sensing data

The study area was remotely observed using both optical and SAR data. SAR data were obtained from the Sentinel-1 mission, while optical imagery was sourced from the Sentinel-2 mission, both accessed through the Copernicus Open Access Hub.

### Sentinel-1

Sentinel-1, presently, has two polar-orbiting satellite constellations in orbit, namely, Sentinel-1 A (since April 2014) and Sentinel-1B (since April 2016)^36^. Both satellites are equipped with a C-Band SAR sensor, which provides backscatter at a frequency of 5.405 GHz, with an incidence angle ranging between 20° and 45°^37^. Each satellite provides imageries every 12 days with a spatial resolution of 10 m. Sentinel-1 provides both co-polarised (VV) and cross-polarised (VH) backscatter, thus facilitating the estimation of various microwave indices. The backscatter value measured by the sensor is the intensity of the returned signal, which was actively sent to the earth’s surface.

### Sentinel-2

Sentinel-2, also a part of the Copernicus space component, has two polar-orbiting satellite constellations in the same sun-synchronous orbit phased 180° apart. The twin satellites, Sentinel-2 A and Sentinel-2B, were launched in June 2015 and March 2017, respectively. Equipped with a Multispectral Instrument (MSI), a passive sensor, Sentinel-2 captures multispectral data across 13 bands^38^. The satellites deliver high spatial resolution data (10–60 m), with three product types viz. level-1B, level-1 C and level-2 A. Level-2 A is the bottom-of-atmosphere (BOA) reflectance in cartographic geometry. In this study, Sentinel-2 level-2 A data were used to supplement Sentinel-1 SAR data.

Analysis was performed on both Sentinel-1 and Sentinel-2 data during the summer rice cultivation season. However, during the *kharif* season, only Sentinel-1 data was analyzed due to heavy cloud cover and the lack of cloud-free optical images. Sentinel-1 SAR data was also pre-processed in Sentinel Application Platform (SNAP) toolbox version 8.0.0 before deriving SAR indices. The pre-processing steps involved applying a precise orbit file, followed by radiometric calibration to derive the backscattering coefficient sigma nought (σ₀). Next, the refined Lee speckle filtering method was applied^39^ to reduce noise. Geometric distortions in the despeckled images were then corrected using Range Doppler Terrain Correction. Given that the backscattering coefficient (σ₀) has a wide dynamic range, it was converted to decibels (dB). The dB scale, a logarithmic unit, simplifies the representation by compressing the dynamic range into a more manageable format, which is calculated using the formula as follows^40^:

$$dB = 10 \log_{10}({I/I_{0}})$$

where, I = radar return from the target. I_0_ = radar signal incident on the target.

### Rice yield prediction using ML models

Nine machine-learning models including Elastic net (ELNET^41^), Random forest (RF^42^), Multivariate adaptive regression splines (MARS^43^), Support vector regression (SVM^44^), Linear regression with stepwise selection (LRSS^45^), K-nearest neighbours (KNN^46^), eXtreme gradient boosting (XGB^47^), Neural network (NNET^48^) and Cubist^49^ were utilized with remote sensing and biophysical parameters as input for rice yield prediction. We used the remote sensing-derived parameters/indices and observations of biophysical parameters collected at three growth stages (45, 60 and 90 DAT) for predicting pre-harvest rice yield using ML models at 45, 60 and 90 DAT of both summer and *kharif* rice.

### Input parameter for ML models

#### SAR parameters

The four SAR input parameters utilized in this study were VV, VH, VV/VH ratio and Normalized Ratio Procedure Between Bands (NRPB). VV (Vertical-Vertical Polarization) radar signal is both transmitted and received vertically. VV backscatter is sensitive to surface roughness and moisture content^50^. On the other hand, VH (Vertical-Horizontal Polarization) radar signal is transmitted vertically and received horizontally. VH backscatter is often sensitive to volume scattering from vegetation or biomass^51^. VV/VH ratio enhances the discrimination between different land cover types^52^. The NRPB using VV & VH was calculated to produce a larger set of covariates for the model inputs and to assist in yield prediction. It may provide additional insights into crop growth and help improve the accuracy of yield predictions when combined with the remote sensing indices and machine learning techniques^53^.

#### Optical parameters

In this study, four optical indices were employed as input parameters for yield prediction: the Normalized Difference Vegetation Index (NDVI), the Red Edge Normalized Difference Vegetation Index (RENDVI), the Ratio Vegetation Index (RVI), and the Land Surface Water Index (LSWI). NDVI is widely used to assess vegetation health and biomass, providing key insights into crop vigour and potential yield^53,54^. RENDVI enhances the sensitivity to chlorophyll content and early stress detection, offering a more detailed view of crop conditions crucial for accurate yield prediction^55–58^. RVI, by comparing NIR to red band reflectance, complements NDVI by providing additional information on plant canopy structure and density^59–61^. LSWI, which utilizes NIR and SWIR bands, is crucial for evaluating vegetation moisture levels and enhancing yield predictions^62,63^. All these optical and SAR parameters/indices were chosen because they provide complementary information about vegetation properties, enhancing the overall yield prediction analysis.

#### Summer rice

In summer season, eight remote sensing parameters and five biophysical parameters at each stage were used as input data to train all the ML models. Out of eight remote sensing parameters used, four are SAR parameters (VV, VH, VV/VH ratio and NRPB) and four are optical parameters (NDVI, RENDVI, RVI and LSWI). The five biophysical parameters, namely DBM, FBM, MC, LAI, and plant height, were used in training the ML models. We collected 36 data points of each input parameter at each stage.

#### Kharif rice

**Fig. 2:**
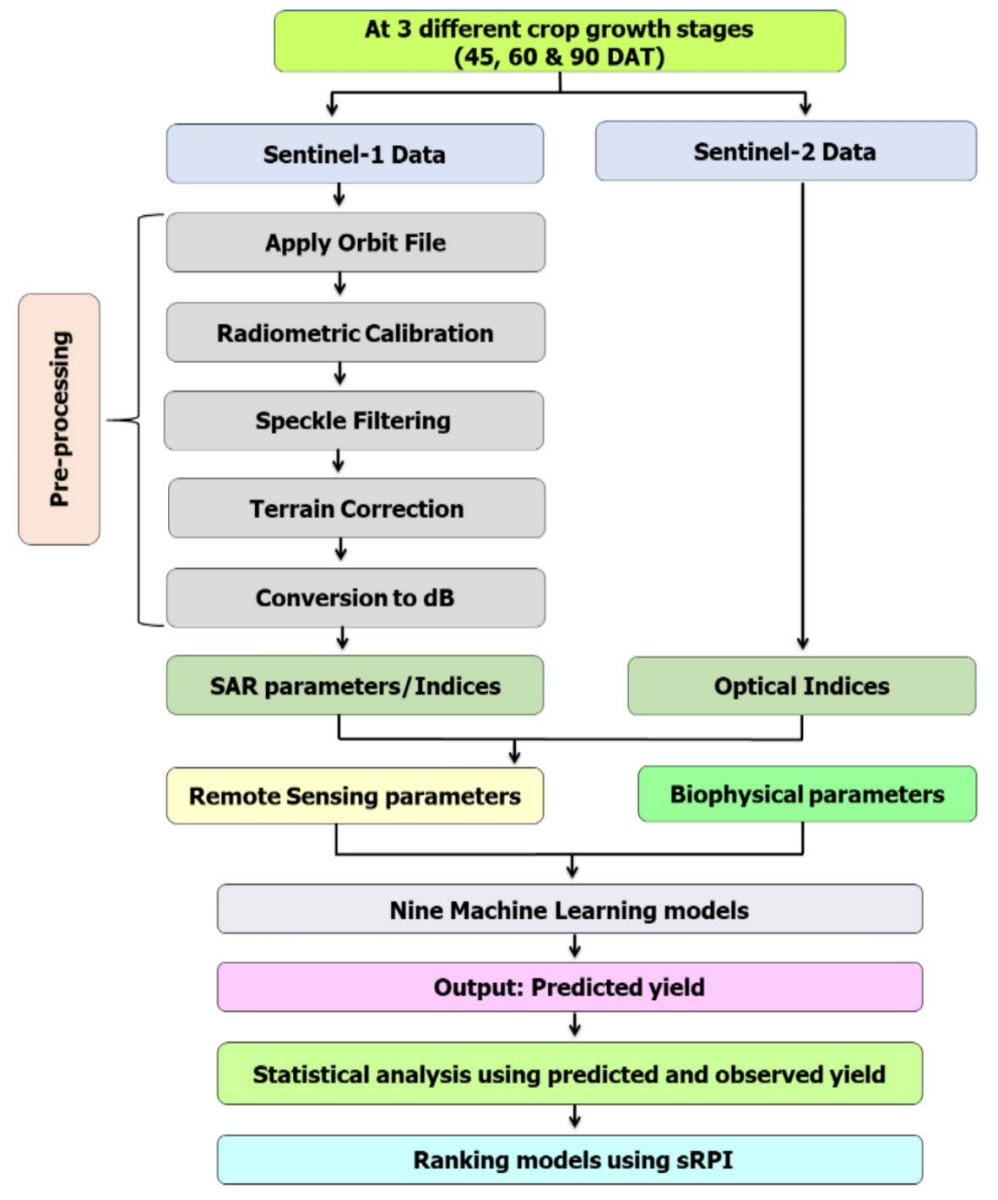
Flow chart describing the methodology of the study in brief.

Unlike in summer rice, only four remote sensing parameters were used for *kharif* rice. These four parameters were all SAR parameters viz. VV, VH, VV/VH ratio and NRPB. However, no optical parameter was used due to the unavailability of cloud-free optical images during the *kharif* season. The biophysical parameters remained the same as that of summer rice, viz. DBM, FBM, MC, LAI and plant height. We collected 64 data points of each input parameter at each stage. The methodology followed for yield prediction is presented in Fig. 2.

### Training, testing and evaluation of machine learning models

Before being used as inputs for the machine learning models, the values of each input parameter (predictor variables) were normalized. The normalized input parameters were divided into a 70:30 ratio, with 70% of the data used for training the models and the remaining 30% used for testing the models^64,65^. The machine learning models were optimized and trained using a ten-fold cross-testing with five repetitions, utilizing the ‘caret’ package^66^ of R software version 4.2.2^67^. For ML models, hyperparameters are important configuration variables set before training begins. They control the learning process and determine the values of model parameters that the learning algorithm will ultimately learn. Hyperparameter tuning can significantly impact a model’s accuracy, generalization, and other metrics, and hence, proper tuning of these hyperparameters is essential for optimizing the model’s performance^68^. Techniques like grid search and random optimization are commonly used to explore and identify the best hyperparameter settings. Random optimization determines hyperparameters by randomly sampling from a predefined range of values. Unlike grid search, which exhaustively evaluates every possible combination, random optimization selects random combinations and evaluates them and hence can be more efficient and effective, especially when dealing with a large number of hyperparameters or a vast search space^69^. Therefore, in this work, hyperparameter tuning was performed using random optimization, with the Root Mean Squared Error (RMSE) values evaluated throughout the optimization process to assess performance. The values of hyperparameters were evaluated and the set that resulted in the lowest RMSE value during the training phase of the model was selected^70^. The prediction ability of the models and performance of model combination approaches was assessed using the coefficient of determination (R^2^^71^ ), Willmott index of agreement (d-index^72^ ), mean bias error (MBE^71^ ), and root mean squared error (RMSE^71^).

We employed the Standardized Ranking Performance Index (sRPI) for an overall assessment of a model’s performance by combining results from training and testing using multiple statistical criteria. The sRPI is calculated by normalizing performance values of statistical criteria to a range of 0 to 1, where 1 indicates the best performance and 0 the worst. This involves a two-step transformation: scaling values directly for criteria where higher is better (R^2^ and d-index), and inverting and scaling where lower is better (RMSE and MBE). The normalized values are averaged to compute each model’s initial ranking performance index (RPI). The sRPI is then derived by standardizing these RPI values across all scenarios, ensuring a relative comparison of models’ performance^73^.

### Data analysis

MS Excel and R software version 4.2.2 were utilized for conducting all necessary statistical analysis. The scripts were designed and run in the software. ArcGIS version 9.1, Google Earth Pro, SNAP version 8.0.0 and ENVI version 4.7 were also utilized for analysing satellite data. The overall methodology of this study is depicted in Fig. 2.

## Results

### Performances of ML models to predict summer rice yield at 45 DAT

**Fig. 3:**
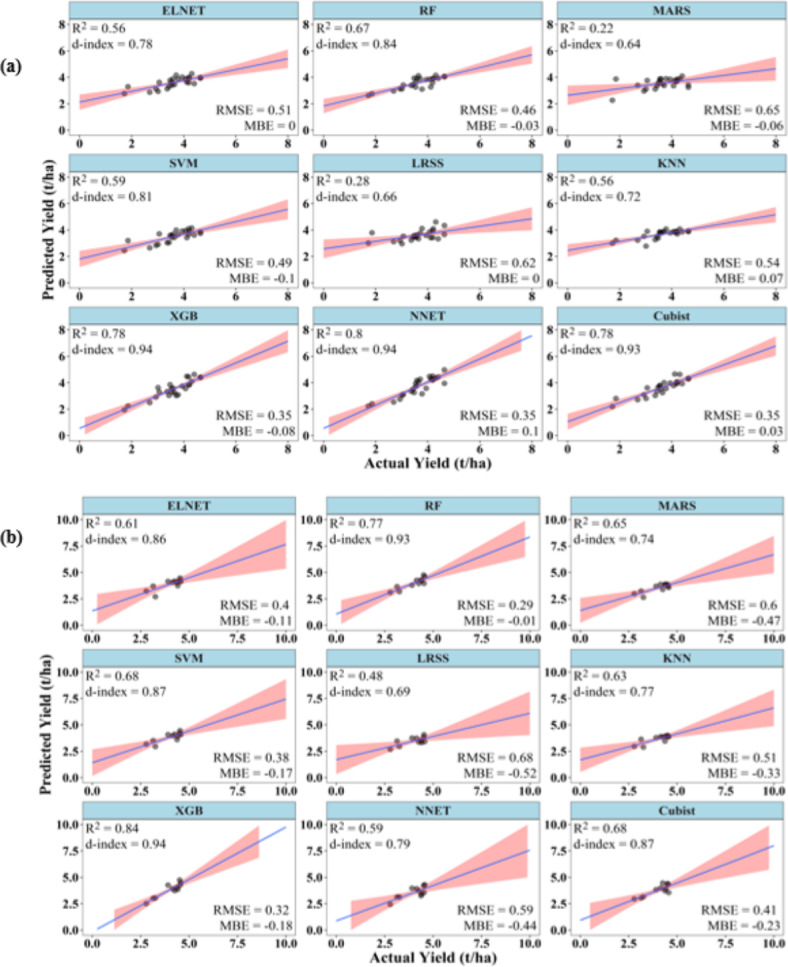
Performance of ML models during (**a**) training and (**b**) testing to predict summer rice yield at 45 DAT.

At 45 DAT, the ML models were able to predict the summer rice yield with acceptable accuracies. During training, Cubist, NNET and XGB have R^2^ and d-index above 0.75 and 0.90, respectively, while their RMSE remained below 0.36 t/ha. NNET has the best values of R^2^ (0.80). The d-index of NNET and XGB were equal (0.94) and highest among all the ML models. The RMSE of XGB, NNET and Cubist were also lowest and equal (0.35 t/ha). The MARS model has the lowest R^2^ and d-index values of 0.22 and 0.64, respectively. MARS also has the highest RMSE of 0.65 t/ha (Fig. 3). MBE ranged between ± 0.10 t/ha for all the models.

During testing, XGB and RF have R^2^ above 0.75. The d-index varied between 0.69 and 0.94, with XGB registering the highest d-index value (0.94), followed by RF (0.93). RMSE of the models ranged between 0.29 and 0.68 t/ha, with the lowest RMSE value shown by RF (0.29 t/ha) followed by XGB (0.32 t/ha). LRSS has the lowest R^2^ and d-index values of 0.48 and 0.69, respectively, and it also has the highest RMSE of 0.68 t/ha. The MBE varied between − 0.01 and − 0.52 t/ha, which indicates under-prediction by the ML models during the testing stage. RF had the lowest, while LRSS had the highest MBE (Fig. 3).

### Performances of ML models to predict summer rice yield at 60 DAT

**Fig. 4:**
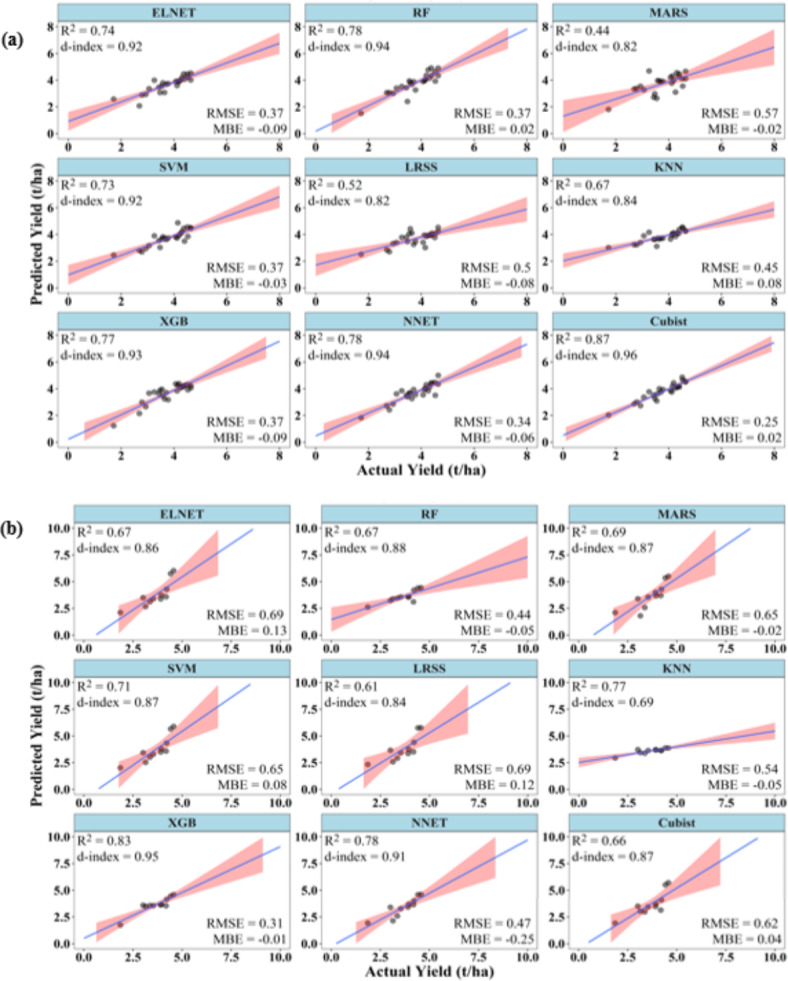
Performance of ML models during (**a**) training and (**b**) testing to predict summer rice yield at 60 DAT.

At 60 DAT, the ML models were able to predict the rice yield with improved accuracies. During training, Cubist, NNET, RF, XGB, ELNET, and SVM have R^2^ and d-index above 0.70 and 0.90, respectively. All these six models also have RMSE values below 0.4 t/ha. The MBE of all the models varied between ± 0.09 t/ha. Cubist has the best values of R^2^ (0.87), d-index (0.96), and RMSE (0.25 t/ha). On the contrary, MARS model has the poorest values of R^2^ (0.44), d-index (0.82) and RMSE (0.57 t/ha) (Fig. 4).

During testing, though SVM, KNN, XGB and NNET showed R^2^ above 0.70, only XGB and NNET showed d-index above 0.90. The XGB model has the highest R^2^ (0.83), d-index (0.95) and lowest RMSE of 0.31 t/ha. All other models showed RMSE higher than 0.4 t/ha, with ELNET and LRSS showing the highest RMSE of 0.69 t/ha. LRSS also has the lowest R^2^ of 0.61, while KNN has the lowest d-index value (0.69). The MBE of all the models varied between − 0.25 and 0.13 t/ha (Fig. 4).

### Performances of ML models to predict summer rice yield at 90 DAT

**Fig. 5:**
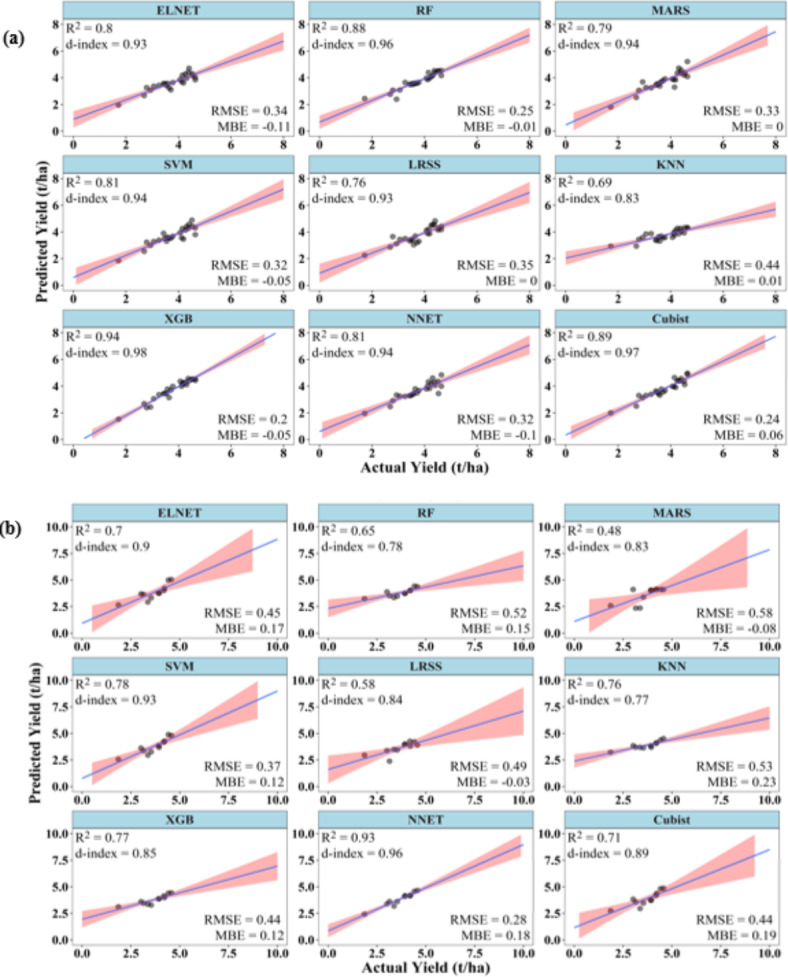
Performance of ML models during (**a**) training and (**b**) testing to predict summer rice yield at 90 DAT.

At 90 DAT, ML models were able to predict rice yield with accuracies better than at 45 and 60 DAT. During training, except KNN, all models had R^2^ above 0.75, d-index above 0.90, and RMSE below 0.35 t/ha. XGB, Cubist and RF have R^2^ above 0.85, d-index above 0.95, and RMSE below 0.25 t/ha. XGB has the best values of R^2^ (0.94), d-index (0.98), and RMSE (0.2 t/ha). Similarly, KNN has the poorest values of R^2^ (0.69), d-index (0. 83), and RMSE (0.44 t/ha). The MBE of all the models varied between − 0.11 and 0.06 t/ha (Fig. 5).

During testing, NNET, SVM, XGB, and KNN had R^2^ above 0.75, whereas only NNET and SVM had d-index above 0.9. The models NNET and SVM also showed RMSE below 0.4 t/ha. The MBE of all the models was positive except for LRSS and MARS. The MBE varied from − 0.08 to 0.23 t/ha. NNET has the best values of R^2^ (0.93), d-index (0.96), and RMSE (0.28 t/ha). On the other hand, MARS has the lowest R^2^ (0.48) and highest RMSE (0.58 t/ha). KNN has the lowest d-index value of 0.77 (Fig. 5).

### Ranking of ML models for predicting the yield of summer rice using sRPI values

**Fig. 6:**
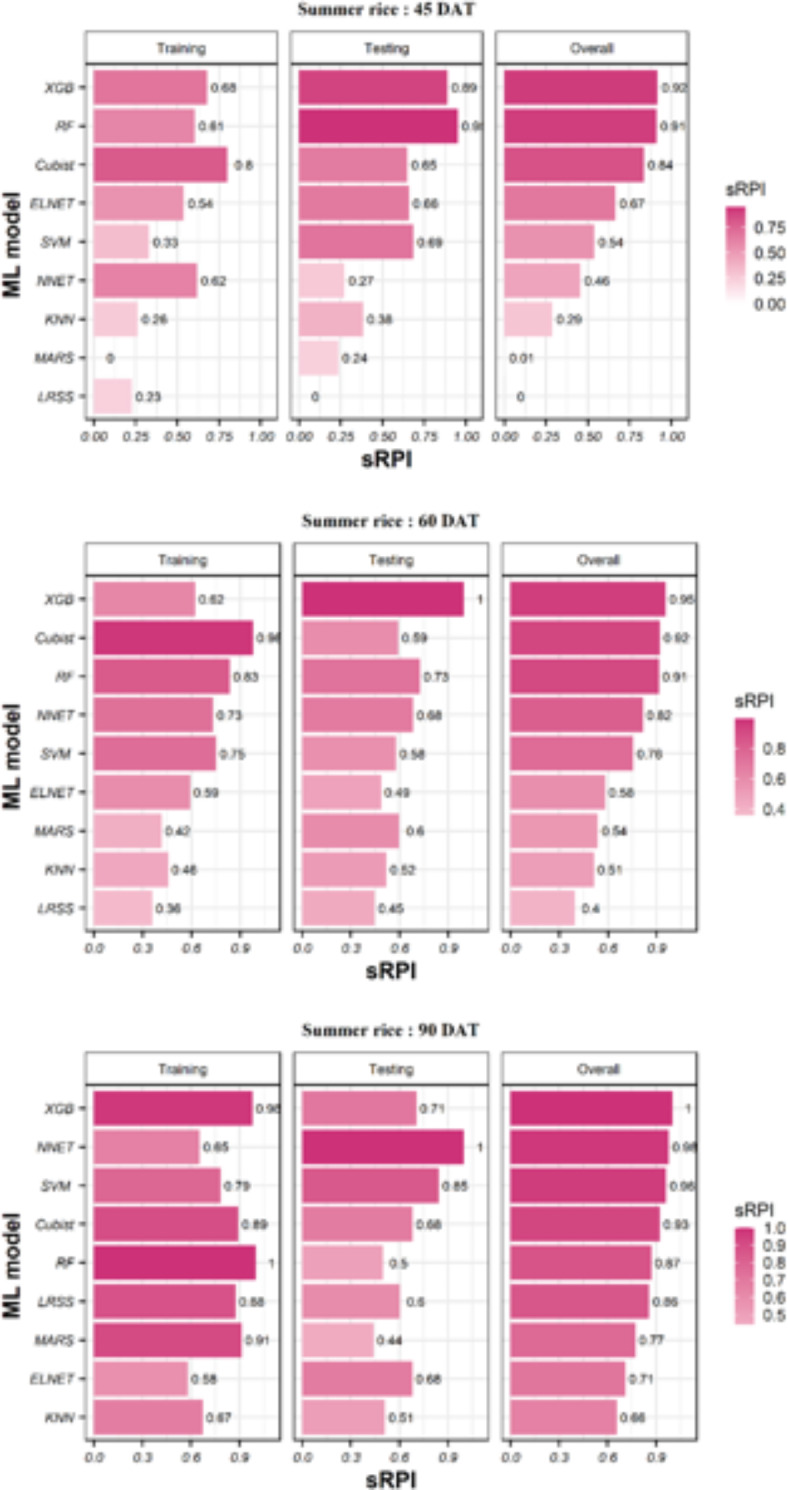
Stage-wise ranking of ML models using the sRPI values for predicting summer rice yield.

At 45 DAT, the sRPI indicated that Cubist and RF were the best-performing models during training and testing, respectively. Overall, XGB was the best, and LRSS was the poorest performing model in predicting summer rice yields at 45 DAT. At 60 DAT, the sRPI indicated that Cubist and XGB were the best-performing models during training and testing, respectively. Overall, XGB was the best-performing model in predicting summer rice yields at 60 DAT. On the other hand, LRSS was the poorest performing model during training, testing and overall performance. At 90 DAT, the sRPI indicated that RF and NNET were the best-performing models during training and testing stages, respectively. Similarly, ELNET and MARS were the poorest performing models during training and testing, respectively. Overall, XGB was the best, and KNN was the poorest-performing model in predicting rice yields at 90 DAT (Fig. 6).

**Fig. 7:**
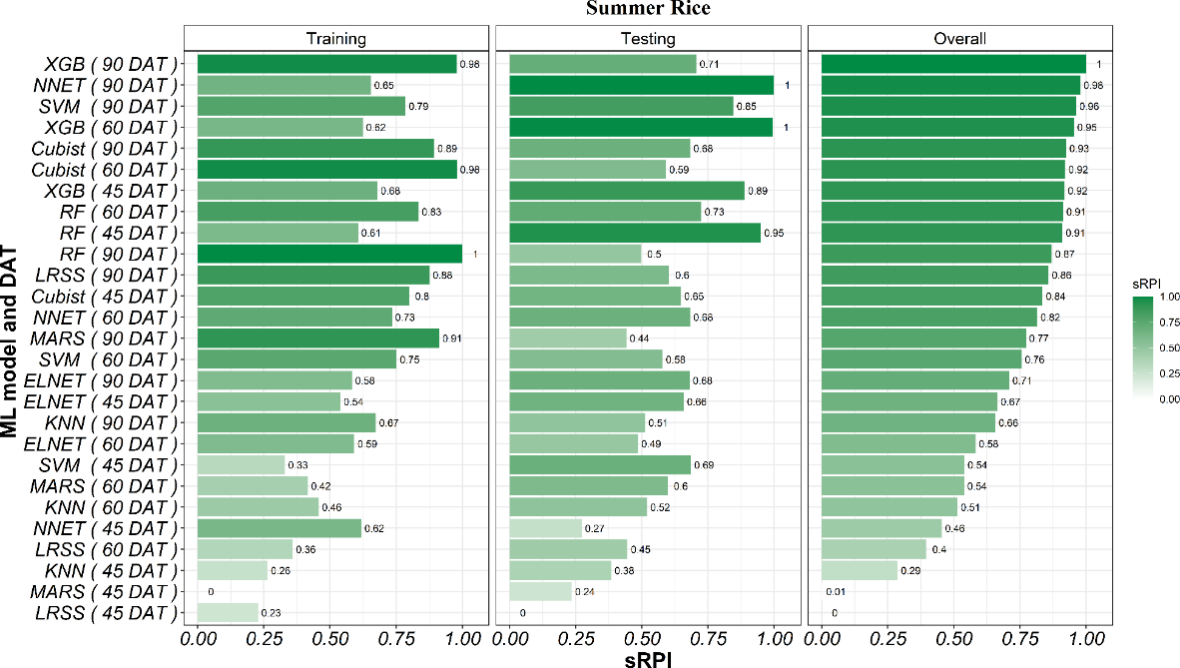
Ranking of ML models using the sRPI values for predicting summer rice yield.

The sRPI rankings of all three stages and nine ML models combination (3 × 9) indicated that at 90 DAT RF was the best-performing ML model and dataset combination during training, while MARS at 45 DAT was the poorest. During testing, NNET at 90 DAT data and XGB at 60 DAT data performed equally well in terms of statistics we used in our work, while LRSS at 45 DAT showed lowest performance. However, the overall ranking obtained by considering both training and testing indicated that XGB model was the best for predicting rice yield at 90 DAT, followed by NNET and SVM. On the other hand, LRSS at 45 DAT achieved the poorest overall ranking for predicting rice yield The detailed ranks of all the models and data pairs can be seen in Fig. 7. The hyperparameters and the important variables of best models for predicting summer rice yield based on sRPI ranking are presented in Supplementary Table 1.

### Performances of ML models to predict kharif rice yield at 45 DAT

**Fig. 8:**
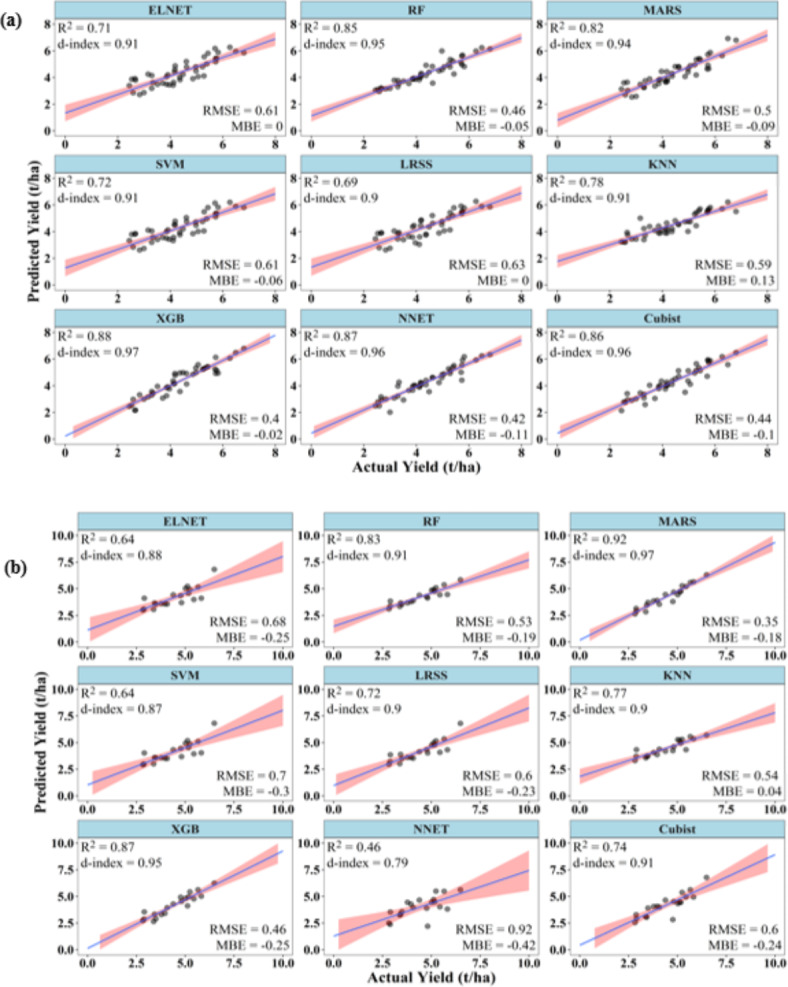
Performance of ML models during (**a**) training and (**b**) testing to predict *kharif* rice yield at 45 DAT.

At 45 DAT, the ML models were able to predict the *kharif* rice yield with acceptable accuracies. During training, except for ELNET, SVM, and LRSS, all other models had R^2^ above 0.75. However, XGB showed the best R^2^ (0.88), d-index (0.97) and RMSE (0.4 t/ha) values. LRSS showed the lowest R^2^ (0.69) and, d-index (0.90) and highest RMSE (63 t/ha) values. The MBE for all the models varied between − 0.11 and 0.13 (Fig. 8).

During testing, except for ELNET, SVM, and NNET, all other models had R^2^ above 0.70. All the models, except NNET, showed a d-index above 0.85. However, in the case of RMSE, Except MARS, all other models showed values above 0.4 t/ha. Among all the models, MARS performed best during training, with the highest values of R^2^ (0.92), d-index (0.97), and the lowest value of RMSE (0.35 t/ha). NNET has the poorest performance with the lowest values of R^2^ (0.46), d-index (0.79), and highest RMSE (0.92 t/ha) (Fig. 8).

### Performances of ML models to predict kharif rice yield at 60 DAT

**Fig. 9:**
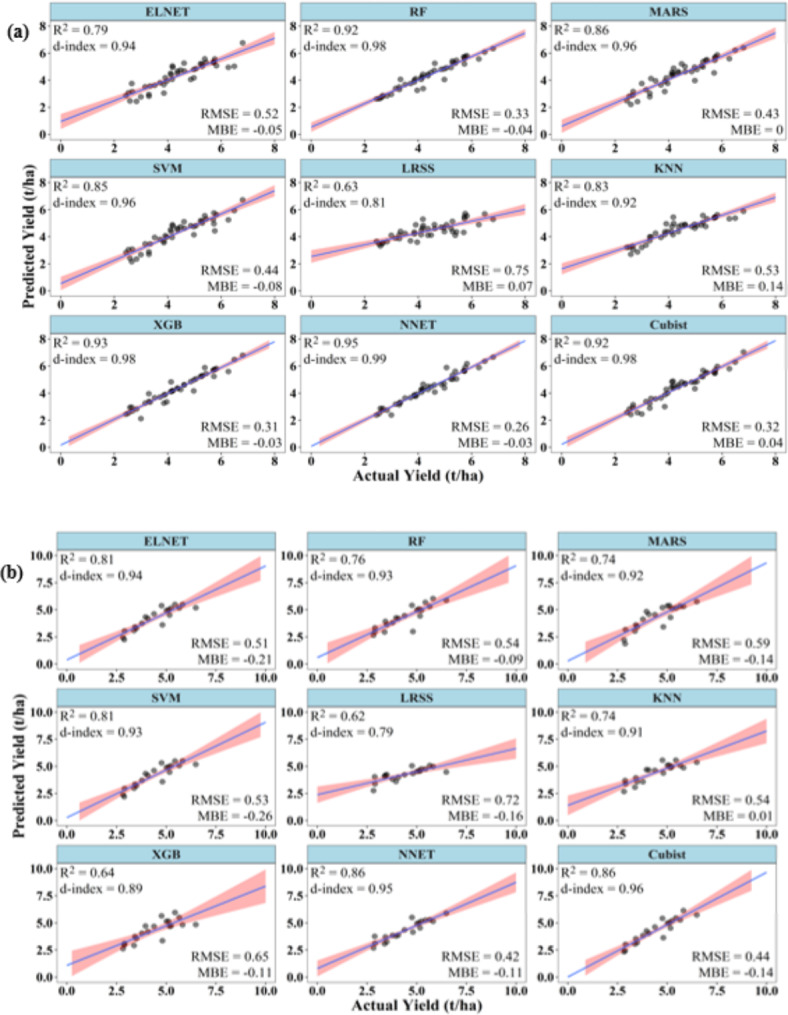
Performance of ML models during (**a**) training and (**b**) testing to predict *kharif* rice yield at 60 DAT.

Again, at 60 DAT, the ML models were able to predict the *kharif* rice yield with improved accuracies. During training, except LRSS, all ML models have R^2^ above 0.75 and d-index above 0.9. In case of RMSE, it varied from 0.26 to 0.75 t/ha. Except LRSS, ELNET and KNN, all other ML models had RMSE values lower than 0.5 t/ha. The model NNET had the best combination of high values of R^2^ (0.95), d-index (0.99) and RMSE (0.26 t/ha), while LRSS had the lowest values of R^2^ (0.63), d-index (0.81) and high RMSE (0.75 t/ha). The MBE varied between − 0.08 and 0.14 t/ha. The R^2^, d-index and RMSE of NNET, XGB, RF and Cubist were almost similar during the training phase (Fig. 9).

During testing, except LRSS and XGB, all other ML models had R^2^ and d-index above 0.70 and 0.9, respectively. Cubist and NNET have the highest R^2^ values of 0.86. Cubist has highest d-index (0.96) very closely followed by NNET (0.95). NNET has the lowest RMSE value of 0.42 t/ha. LRSS has the poorest R^2^ (0.62), d-index (0.79) and RMSE (0.72 t/ha). The MBE again remained negative for all the models except KNN during the testing phase, indicating an underestimation of the yield. The MBE of all the models varied between − 0.26 and 0.01 t/ha (Fig. 9).

### Performances of ML models to predict kharif rice yield at 90 DAT

**Fig. 10:**
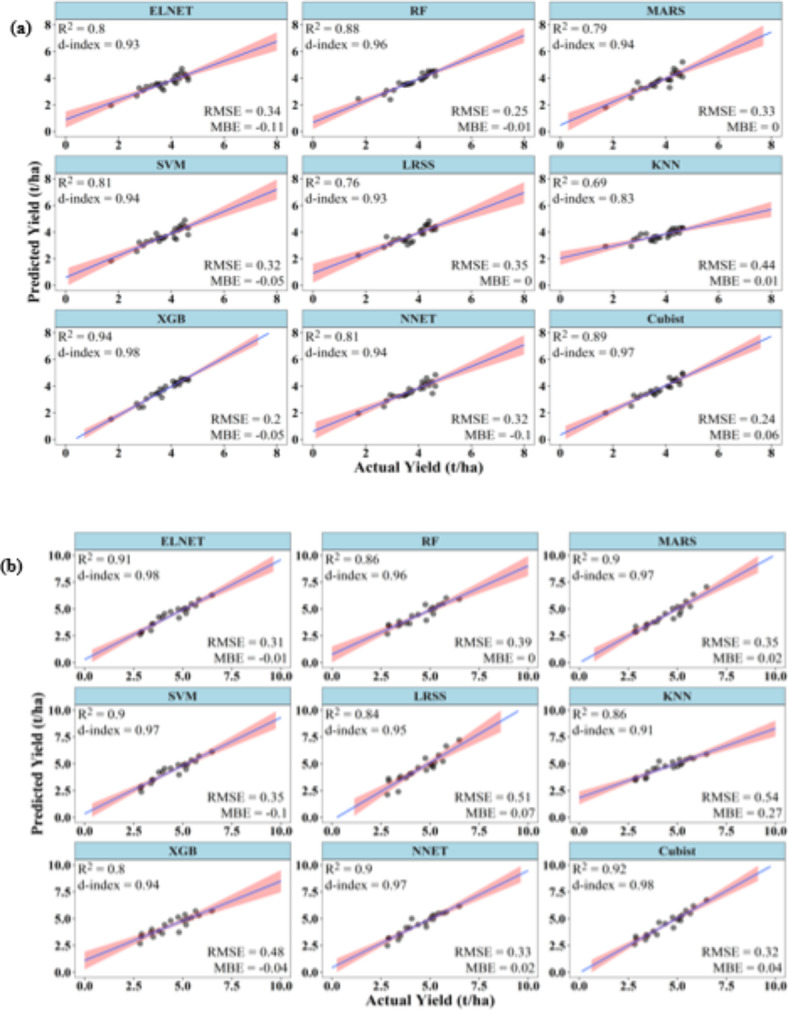
Performance of ML models during (**a**) training and (**b**) testing to predict *kharif* rice yield at 90 DAT.

As in case of summer rice, in *kharif* rice also ML models at 90 DAT were able to predict the yield with accuracies better than at 45 and 60 DAT. During training, except KNN, all ML models have R^2^ above 0.75 and d-index above 0.90. XGB has the highest R^2^ value of 0.94, d-index of 0.98 and lowest RMSE of 0.2 t/ha. On the other hand, KNN has the lowest R^2^ values of 0.69, d-index of 0.83 and highest RMSE of 0.44 t/ha. The MBE varied between − 0.11 and 0.06 t/ha (Fig. 10).

During testing, all ML models have R^2^ equal to or above 0.80. The d-index of every ML model was above 0.90. Cubist has the highest R^2^ value of 0.92, whereas XGB has the lowest R^2^ values of 0.80. Cubist and ELNET have highest and equal d-index value of 0.98 followed by NNET, MARS and SVM with d-index value of 0.97. The lowest d-index of 0.94 was recorded for XGB. The RMSE of all ML models were below 0.4 t/ha, except KNN, LRSS and XGB. The model ELNET had the lowest RMSE value of 0.31 t/ha, whereas KNN has the highest RMSE of 0.54 t/ha. The MBE varied between − 0.10 and 0.27 t/ha. KNN has the maximum value of MBE (Fig. 10).

### Ranking of ML models for predicting the yield of kharif rice using sRPI values

**Fig. 11:**
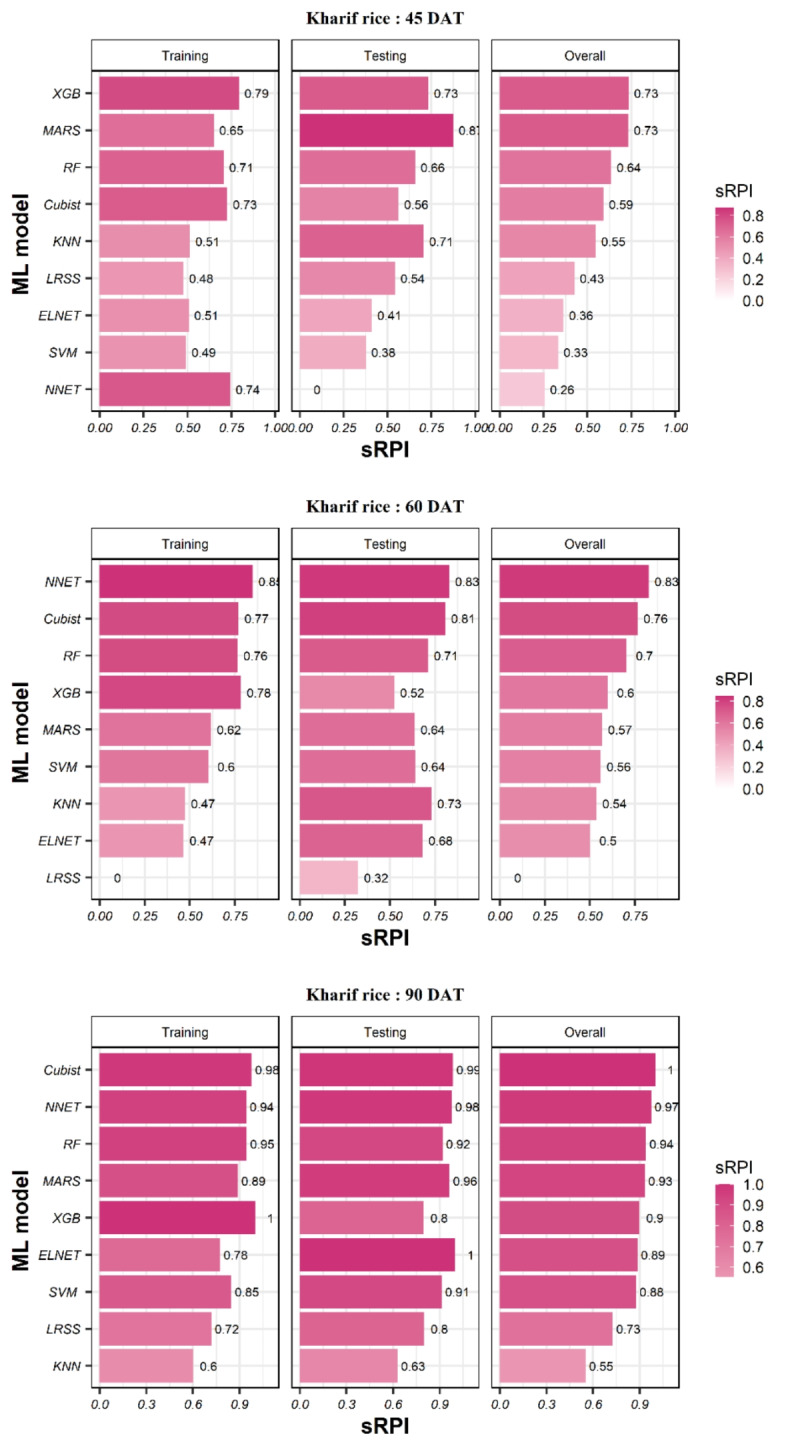
Stage-wise ranking of ML models using the sRPI values for predicting *kharif* rice yield.

At 45 DAT, the sRPI indicated that XGB and MARS were the top performing models at training and testing, respectively, while LRSS and NNET were the poorest performing models at training and testing stages, respectively. Overall, XGB and MARS performed equally well and NNET was the poorest-performing model in predicting *kharif* rice yields at 45 DAT. At 60 DAT, sRPI indicated that NNET was the best-performing model at both the training and testing stages. LRSS was the poorest-performing model during both training and testing. Hence, the overall sRPI also indicated NNET as the best and LRSS as the poorest performing model in predicting *kharif* rice yields at 60 DAT. At 90 DAT, the sRPI indicated that XGB and ELNET were best best-performing models during the training and testing stages, respectively. Similarly, KNN was the poorest-performing model during both training and testing. Overall, Cubist was the best and KNN was the poorest performing model in predicting *kharif* rice yields at 90 DAT (Fig. 11).

**Fig. 12:**
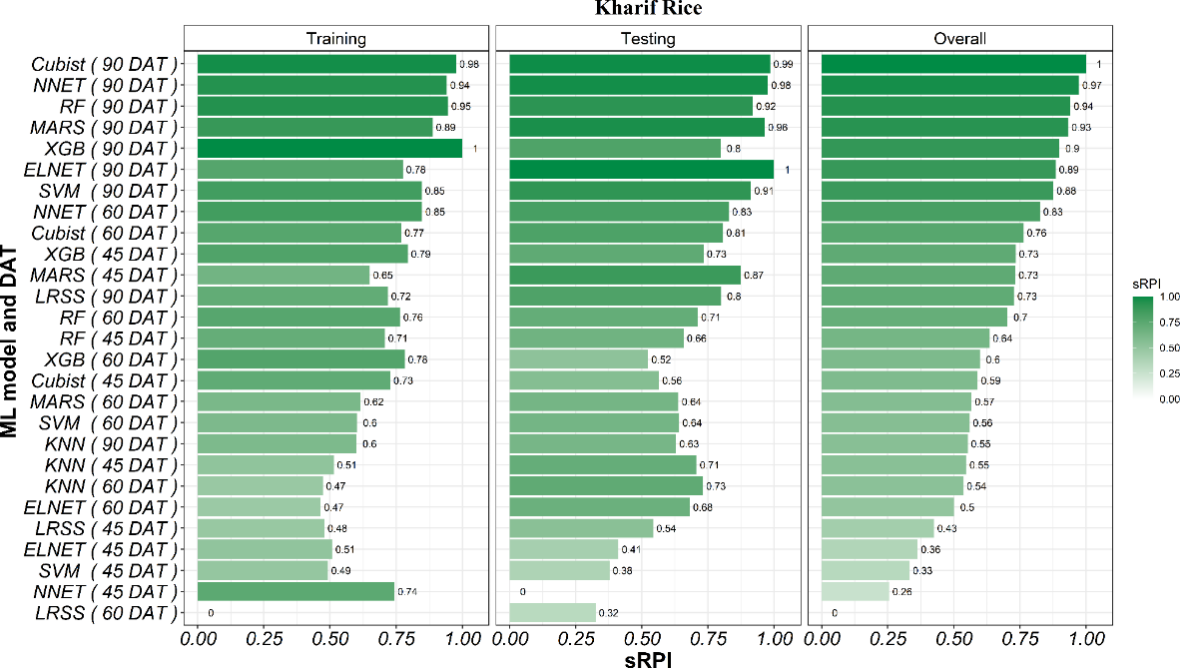
Ranking of ML models using the sRPI values for predicting *kharif* rice yield.

The sRPI rankings of all the three stages and nine ML model combinations (3 × 9) indicated that at 90 DAT Cubist was the best-performing ML model during training, while KNN and ELNET at 60 DAT were the poor performers. During testing, ELNET at 90 DAT was the best performing model, while NNET at 45 DAT showed lowest performance. However, the overall ranking obtained by considering both training and testing indicated that Cubist model was the best for predicting rice yield at 90 DAT, followed by NNET and RF. On the other hand, LRSS at 60 DAT achieved poorest overall ranking for predicting rice yield. The detailed ranks of all the models and data pairs can be seen in Fig. 12. The hyperparameters and the important variables of best models for predicting *kharif* rice yield based on sRPI rankings is presented in Supplementary Table 2.

## Discussion

This research set out to predict rice yield in both summer and *kharif* seasons through the integration of optical and SAR data with crop biophysical parameters using machine learning models. The ML models successfully made accurate yield predictions for both summer and *kharif* rice using remote sensing parameters/indices and biophysical parameters from ground-based measurements as training data. Our findings showed that the values of model performance metrics (R^2^, d-index, RMSE, and MBE) obtained by ML models for predicting rice yield were close to their critical values as compared to other yield prediction models using only remote sensing data. It is also noteworthy to observe that though predictions made closer to harvest time are more accurate, the initial predictions made at an earlier stage, despite having lower accuracy, are still more valuable for advance decision-making.

Several research studies support our findings and predicted rice yield using different input datasets with machine learning models and reported that rice yield can be predicted with considerable accuracies (R^2^ > 0.7 and RMSE < 0.7 t/ha)^19,30,33,34,74,75^. The ML models have been shown to be more effective in predicting yields than traditional statistical methods due to their ability to identify complex patterns and relationships in data, allowing for more comprehensive analysis^76^. However, the model performance metrics values observed in our study are far better than those of previous studies. For instance, Abbas et al.^24^ predicted potato yield using soil parameters and NDVI data with SVM, ELNET, KNN, and simple regression models, achieving an RMSE of 4.62 t/ha and an R² of 0.70, which is lower as compared to our results. Schwalbert et al.^77^ integrated MODIS optical data with meteorological parameters to predict soybean yield using multivariate OLS, RF and LSTM-based neural networks and was able to achieve RMSE of 0.39 and 0.32 Mg/ha with RF and LSTM-based neural networks. Studies that used Sentinel-2 data for crop yield prediction with machine learning often reported results that are either lower or comparable to ours, depending on the crop, season, and methodology employed. In rice, Nazir et al.^78^ reported R^2^ = 0.84 and RMSE = 0.12 t/ha using Sentinel-2 derived indices. Gómez et al.^79^ reported R^2^ of 0.93 for potato yield prediction using Sentinel-2 data with ML models. Similarly, Bebie et al.^80^, utilizing Sentinel-2 images, predicted the yield of durum wheat and reported R^2^ higher than 0.87 with RF and KNN.

The better model performance metrics/indices in the present study are most likely due to the combined approach of inclusion of multi-sensor data as well as ground-based biophysical parameters of the crop as predictors, which strongly correlate with the overall plant condition, enhancing the accuracy of model predictions. These findings are supported by several researchers who reported improved yield predictions by ML models by the inclusion of biophysical parameters in crops like wheat^81,82^, soybean, corn, and canola^83^, maize^84^ etc. Comparing *kharif* and summer rice prediction in the present study, we observed that the best-performing models have slightly better model performance metrics in *kharif* as compared to summer predictions, which is mostly attributed to the fewer data points available in the summer season for training the ML models.

The superior performance of XGB, Cubist, and NNET in the intricate task of crop yield prediction is attributed to their ability to handle complex non-linear relationships, adaptability to various data structures, effective feature selection, robustness in high-dimensional data, and advanced regularization techniques. In comparison, ELNET, RF, SVM, LRSS, and MARS are relatively weaker models for complex non-linear datasets^85^. These findings are in line with previous studies on yield predictions using these models^86–88^. XGB, Cubist, and RF are decision tree-based methods. Among them, XGB is the most advanced in system and algorithm optimization^89,90^ and hence usually performs better than other ML models. Cubist is similar to RF but incorporates an additional regression at the leaf nodes, enabling it to perform better than RF^91^. The consistent poor performance of KNN over other models is attributed to its high sensitivity for irrelevant features^92^. All of these ML models are compared together in very few studies. For example, Singh et al. worked on the prediction of disease severity in wheat^70^ and chickpea^64^ crops using the XGB, RF, Cubist, SVM, KNN, LRSS, and MARS. They also reported XGB and Cubist as the best ML models using the sRPI rankings, which supports the rankings of the ML models in the present study. Singha et al.^21^ also found Cubist and XGB as superior over RF, SVM, and KNN, while predicting apple yield using ML models and Google Earth engine images.

Furthermore, the present study explored the sensitivity of the yield predictions by ML models to the prediction date, considering that the importance of yield prediction lies in balancing its accuracy with the timing of the prediction. Some previous studies reported a trade-off between the accuracy and prediction date^93,94^. A noteworthy observation from the study is that the yield predictions made closer to harvest time (90 DAT) are more accurate than the predictions made at an earlier stage (45 DAT). Despite having lower accuracy, early-stage predictions provide sufficient lead time for decision-making^81^. In a similar recent study, Singh et al.^70^ demonstrated that incorporating crop biophysical parameters as predictors, alongside remote sensing data, significantly improved the accuracy of ML models in predicting wheat yield under varying levels of yellow rust severity. Improved accuracy in prediction was observed with the delay in forecasting date towards harvest. Although this study was conducted in a smaller area in the *Terai* regions of India, the approach outlined here has the potential to be applied to other regions worldwide for early rice yield predictions. Future research should investigate the potential of deep learning techniques and the integration of climate and soil data to improve the accuracy of rice yield predictions.

## Conclusions

The present study developed field-scale rice yield prediction models by integrating remote sensing data (optical and SAR parameters/indices) and ground-based crop biophysical parameters using ML models for both summer and *kharif* rice. The findings demonstrate that the ML models developed for predicting crop yields showed promising results, with accuracy improving as the harvest time approached. In summer rice, XGB was the best model for yield prediction at all three growth stages, while in *kharif* rice XGB, NNET and Cubist were best models for predicting yield at the crop’s early, mid and late growth stage, respectively. Overall, sRPI rankings indicated that XGB, Cubist and NNET were the three best models to predict rice yield, giving best accuracies at 90 DAT in both summer and *kharif* rice. This study indicates that combining ground-based biophysical data with remotely sensed SAR and optical data using ML models enables accurate and timely rice yield predictions, which has the potential to empower farmers, policymakers, and stakeholders to optimize resource allocation, devise market strategies, and manage risks under changing climatic conditions, ultimately reducing yield failures and food shortages.

## Supplementary Information

Supplementary Material 1

## Data Availability

All data supporting the findings of this study are available within the paper and its Supplementary Information.
