# Supplementary material for: Rice yield prediction through integration of biophysical parameters with SAR and optical remote sensing data using machine learning models
Source: Sci Rep. 2024 Sep 17;14:21674. doi: 10.1038/s41598-024-72624-4 (PMC11408675; doi:10.1038/s41598-024-72624-4)
Supplement: Supplementary file 1 — Supplementary Material 1 [file 41598_2024_72624_MOESM1_ESM.docx]

**Supplementary Table 1**

Hyperparameters and important variables of top 10 models for predicting summer rice yield

| **S.No.** | **Model** | **DAT** | **Hyperparameters** |
| --- | --- | --- | --- |
| 1. | XGB | 90 | nrounds = 50, max_depth = 2, eta = 0.4, gamma = 0, colsample_bytree = 0.8, min_child_weight = 1 and subsample = 1 |
| 2. | NNET | 90 | size = 5 and decay = 1e-04 |
| 3 | SVM | 90 | cost = 1 and Loss = L1 |
| 4 | XGB | 60 | nrounds = 50, max_depth = 2, eta = 0.4, gamma = 0, colsample_bytree = 0.6, min_child_weight = 1 and subsample = 1 |
| 5 | Cubist | 90 | nrounds = 150, max_depth = 2, eta = 0.3, gamma = 0,  colsample_bytree = 0.8, min_child_weight = 1 and subsample = 0.75 |
| 6 | Cubist | 60 | committees = 1 and neighbours = 5 |
| 7 | XGB | 45 | nrounds = 150, max_depth = 1, eta = 0.4, gamma = 0,  colsample_bytree = 0.6, min_child_weight = 1 and subsample = 1 |
| 8 | RF | 60 | mtry = 13, splitrule = extratrees and min.node.size = 5 |
| 9 | RF | 45 | mtry = 7, splitrule = extratrees and min.node.size = 5 |
| 10 | RF | 90 | mtry = 13, splitrule = variance and min.node.size = 5 |

**Supplementary Table 2**

Hyperparameters and important variables of top 10 models for predicting kharif rice yield

| **S.No.** | **Model** | **DAT** | **Hyperparameters** |
| --- | --- | --- | --- |
| 1. | Cubist | 90 | committees = 10 and neighbors = 5 |
| 2. | NNET | 90 | size = 5 and decay = 1e-04 |
| 3 | RF | 90 | mtry = 9, splitrule =extratrees and min.node.size = 5. |
| 4 | MARS | 90 | nprune = 9 and degree = 1 |
| 5 | XGB | 90 | nrounds = 150, max_depth = 2, eta = 0.3, gamma = 0,  colsample_bytree = 0.8, min_child_weight = 1 and subsample = 0.75 |
| 6 | ELNET | 60 | alpha = 0.1 and lambda = 0.001978794 |
| 7 | SVM | 60 | cost = 1 and Loss = L2 |
| 8 | NNET | 60 | size = 5 and decay = 0 |
| 9 | Cubist | 60 | committees = 20 and neighbours = 9 |
| 10 | XGB | 45 | nrounds = 150, max_depth = 2, eta = 0.3, gamma = 0,  colsample_bytree = 0.8, min_child_weight = 1 and subsample = 1 |
